# Case Series of Triathletes with Takotsubo Cardiomyopathy Presenting with Swimming-Induced Pulmonary Edema

**DOI:** 10.1155/2022/3602505

**Published:** 2022-10-26

**Authors:** Caitlin Rigler, Gautam Menon, Samuel Lipworth, Jeremy P Langrish, Courtney Kipps, Mayooran Shanmuganathan, Ralph Smith

**Affiliations:** ^1^Department of Sport and Exercise Medicine, Nuffield Orthopaedic Centre, Oxford University Hospitals NHS Trust, Oxford, UK; ^2^Emergency Department, John Radcliffe Hospital, Oxford University Hospitals NHS Trust, Oxford, UK; ^3^Department of Cardiology, John Radcliffe Hospital, Oxford University Hospitals NHS Trust, Oxford, UK; ^4^Institute of Sport, Exercise and Health, Division of Surgery and Interventional Sciences, University College London, London, UK

## Abstract

**Objectives:**

To report three cases of triathletes who presented with swimming-induced pulmonary edema (SIPE) following water immersion. They were subsequently diagnosed with Takotsubo cardiomyopathy (TCM).

**Design:**

Retrospective case series.

**Method:**

All cases were recreational athletes competing in mass participation triathlons between June 2018 and 2019. They were initially managed by the event medical team and subsequently at the local tertiary level hospital. Written consent was gained from all the subjects.

**Results:**

The three triathletes were aged between 50 and 60 years, two were females, and all presented with acute dyspnoea on exiting the water. Two also presented with chest pain and haemoptysis. A diagnosis of SIPE was suspected by the medical event team on initial presentation of low oxygen saturations and clinical signs of pulmonary oedema. All were transferred to the local emergency department and had signs of pulmonary oedema on chest radiographs. Further investigations led to a diagnosis of TCM with findings of *T* wave inversion in anterolateral electrocardiogram leads and apical hypokinesia on transthoracic echocardiogram and unobstructed coronary arteries.

**Conclusions:**

This case series presents triathletes diagnosed with SIPE and TCM following the open water swim phase. It is unclear whether the myocardial dysfunction contributed to causation of SIPE or was the result of SIPE. Mass participation race organizers must be prepared that both SIPE and TCM can present in this population. Those presenting with an episode of SIPE require prompt evaluation of their cardiac and pulmonary physiology. Further research is required to ascertain the exact nature of the relationship between TCM and SIPE.

## 1. Introduction

Swimming-induced pulmonary edema (SIPE) is a rare cause of life-threatening acute breathlessness occurring during a water-based event. In a four-year prospective cohort study of a large open-water event in Sweden, the incidence was estimated to be 0.44% (95% confidence interval 0.39–0.51%) [1]; other studies have suggested it may be even higher than this [2, 3]. The USA triathlon estimated a total event fatality rate of 1 per 76,000 over a six-year period, with 30/45 (63%) deaths occurring during the swim (though the aetiology of these is not recorded) [4]. Moon et al. found that, of 58 triathlon-related deaths in the USA or Canada, 42 (72.4%) occurred during the swim, and of these, left ventricular hypertrophy (thought to be associated with susceptibility to SIPE), was present in a greater proportion than in the general triathlon population [5]. SIPE is characterized by symptoms and signs of pulmonary oedema following water immersion. A key feature in the majority of cases is the rapid resolution of symptoms within 48 hours [6, 7], and indeed symptoms usually begin to resolve after exiting the water with supportive management.

Whilst the exact pathophysiology remains elusive, a recent study suggests that there is an exaggerated rise in pulmonary artery and pulmonary capillary wedge pressures during exercise in SIPE susceptible individuals [8]. Typical cases of SIPE tend to demonstrate normal underlying cardiac and pulmonary function on further investigation [9]. However, this may be less likely in older swimmer/divers, as an episode of SIPE may unmask other subclinical diseases, particularly cardiac dysfunction [10].

Similar to SIPE, Takotsubo cardiomyopathy (TCM) is an acute and usually reversible form of heart failure presumably triggered by either emotional or physical stressors. However, in contrast, TCM is characterized by acute onset of chest pain, electrocardiogram changes with elevated cardiac enzymes, and a typical echocardiographic pattern involving the left ventricle (apical ballooning and basal hyperkinesis) [11] with the absence of significant atheromatous disease on coronary angiography [12]. Peacher et al. reviewed reported cases of SIPE and highlighted that a significant proportion were found to have cardiac dysfunction on further investigation and highlighted some cases whereby Takotsubo Cardiomyopathy presents as SIPE [10].

This manuscript reports the cases of three triathletes, across two separate events, who developed acute dyspnoea during the swimming part of the event and who presented with pulmonary oedema. They were subsequently diagnosed with TCM upon further investigation. We discuss their clinical presentation, investigation findings, and subsequent management. We explore whether the myocardial dysfunction in these cases contributed to SIPE, or was the result of SIPE. With the growing popularity of water-based events, this report aims to highlight SIPE to race organizers and medical teams; breathless swimmers in whom the diagnosis is considered must have prompt evaluation of their cardiac and pulmonary physiology.

## 2. Materials and Methods

Here, we present a retrospective case series of three recreational triathletes who presented with acute dyspnoea during the swim phase of a mass participation triathlon event in June 2018 (2) and 2019 (1); there were 4862 and 4168 participants in each respective event. All cases were referred to the local emergency department (ED). Medical records from the field, subsequent admission, and follow up were analysed. The subjects were contacted by telephone to clarify any specific details such as self-reported fitness levels and triathlon experience. Informed written consent was gained from all subjects.

## 3. Results

### 3.1. Case 1

A 53-year-old male experienced triathlete developed acute dyspnea and chest pain within 50 metres of starting the 750 m open water swimming phase of a sprint distance triathlon in which he was wearing a tight-fitting wetsuit. The pain was described as “band-like” across the anterior chest, exertional, and nonradiating and was associated with sweating.

Despite worsening symptoms, he completed the rest of the swim phase changing stroke from front crawl to back stroke. He denied swallowing or aspirating water. After exiting the water, on the bike phase, he experienced multiple episodes of haemoptysis, but was determined to finish and walked the final running phase of the event. Upon completion, he sought medical attention and was given GTN spray by the event medical team, which slightly improved hist chest pain.

He was physically fit and had previously completed multiple triathlons including ironman distance. He reported no symptoms in the days preceding the event and felt that he had trained successfully.

On assessment at the triathlon medical tent, his chest pain persisted. He presented with an SpO_2_ of 95% which rose to 99% on 15 L supplementary oxygen. There were no signs of raised jugular venous pressure, and there was no pedal oedema. On chest auscultation, there were bilateral coarse crackles to the mid-zones. Cardiovascular examination was normal with no murmurs heard. Sublingual glyceryl trinitrate (GTN) was administered which relieved his chest pain. He was transferred to the nearest emergency department and given 4 L/min of supplementary oxygen to maintain his saturations.

**Figure 1:**
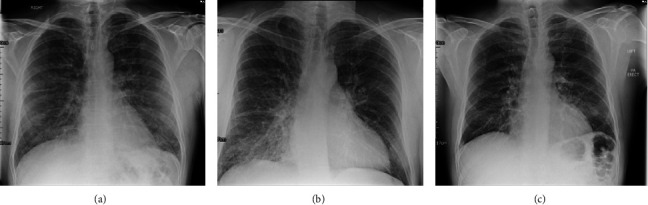
CXR. (a) Case 1 displaying bilateral pulmonary oedema in the hilar region; (b) Case 2 displaying pulmonary oedema with upper zone diversion; (c) Case 3 showing bilateral perihilar interstitial oedema in keeping with evidence of pulmonary oedema.

His further assessment, investigations, and treatment are summarized in Table 1. Initial troponin I value was 0.11 ug/L, rising to 0.15 ug/L at 4 hours. D-Dimer was 1.949 ng/mL. The electrocardiogram (ECG) showed nondynamic *T* wave inversion in leads V4-6 (anterolateral) which had resolved by day two of admission, and the chest X-ray (CXR) demonstrated evidence of pulmonary oedema (Figure 1(a)). Transthoracic echocardiography (TTE) showed an anterior wall motion abnormality with antero-apical hypokinesia. Coronary angiography revealed unobstructed coronary arteries with just minor atheroma in the mid-left anterior descending artery. Hence, a diagnosis of TCM was made.

A single dose of ticagrelor 180 mg was administered as per acute coronary syndrome (ACS) protocol, but was subsequently stopped following the findings during angiography. He was started on daily aspirin 75 mg, atorvastatin 40 mg, and ramipril 1.25 mg and advised against exercise for 6 weeks.

Eight weeks later he was reviewed in the cardiology outpatient department. His exertional dyspnoea had improved since discharged but remained present causing symptoms when walking up hills or stairs (e.g., MRC Dyspnoea Scale Grade 1). He continued to have ongoing chest discomfort, however, this did not clearly and reproducibly occur in an exertional pattern, also occurring apparently randomly at rest. He was thus reassured that this was unlikely to represent ongoing cardiac disease. The ECG confirmed resolution of the previous *T* wave changes and was otherwise unremarkable. A repeat echocardiogram demonstrated good left ventricular systolic function with no regional wall motion abnormality. He was advised to continue taking aspirin, statins, and antihypertensive therapy (given that his blood pressure was at the upper end of normal) as secondary prevention given the incidental finding of coronary atheroma during his coronary angiogram.

### 3.2. Case 2

A 57-year-old female triathlete developed fatigue and dyspnea approximately 300 m into the 750 m swim phase of a triathlon. She found that she was unable to swim any further and had to be rescued from the water by the event safety team. At rest, her dyspnoea persisted but she did not complain of any chest pain, presyncopal symptoms, or palpitations. She had been well in recent days with no cough or fever. She was concerned that she may have inhaled a small volume of water.

Before the triathlon, she swam regularly in a pool and had trained in open water. This was her first triathlon competition and admitted some prerace anxiety regarding this, however, she had completed a 750 m open water swim the preceding weekend.

Past medical history included a previous lumpectomy of the left breast, hysterectomy following an ovarian cyst, and epilepsy for which she was taking lamotrigine (100 mg bd orally); her last seizure was 3 years previously, and she was clear that she had not experienced any seizure activity nor loss of consciousness in the events leading to the current presentation.

On assessment at the medical tent, she was tachypnoeic with a RR of 34 and her SpO_2_ was 78% on RA. This rose to 97% with 15 L supplementary oxygen, but subsequently dropped to 88% when weaning to 4 L oxygen 15 minutes later. She was given 300 mg aspirin and a spray of GTN and taken to ED.

At ED, her oxygen saturation was 94% on RA (Table 1) with bibasal crepitus on auscultation. CXR confirmed pulmonary oedema with upper zone venous diversion (Figure 1(b)). Her ECG showed *T* wave inversion in leads V4–6. Her initial troponin I was 0.26 ug/L and peaked at 1.12 ug/L. She was admitted for further investigations. Echocardiography showed ballooning and akinesis of the left ventricular apex. Subsequent coronary angiography demonstrated angiographically normal coronary arteries, therefore, a diagnosis of TCM was made. She was started on 300 mg aspirin, ramipril 1.25 mg qd, and Bisoprolol 1.25 mg qd.

She was discharged 24 hours later, in the absence of no further symptoms and stable observations. She was advised to refrain from strenuous activities for the next three months. A three-month follow-up appointment was arranged with the consultant cardiologist, although this was later cancelled by the patient.

### 3.3. Case 3

60-year-old female recreational triathlete developed dyspnea and fatigue within 2–3 minutes of the open water swim phase. She wore a tightly fitted wetsuit. With worsening symptoms, she changed stroke from front crawl to breast stroke. She denied any water aspiration. She eventually completed the swim but required assistance to be removed from the water. She had severe dyspnea, developed haemoptysis (a small volume of pink frothy sputum) but no chest pain, and was taken to the event medical team.

This triathlete had not experienced any similar symptoms in the weeks preceding the race and had trained regularly. She had successfully completed six previous open water training swims, stating that she could comfortably swim one mile continuously indoors, although she remarked she did experience mild exertional dyspnea when swimming in open water compared to the swimming pool. She had no other significant medical history and had never smoked.

On assessment at the triathlon medical tent, she denied having chest pain. Her SpO_2_ was 88% on RA, RR was 20, and on chest auscultation, there were bilateral coarse crackles to the mid-zones. Her heart rate was 105 bpm and her blood pressure was within normal range (112/85 mm/Hg). Cardiovascular examination was normal with no murmurs heard. She was started on supplementary oxygen (2 L), and 30 mg of intravenous furosemide was administered. She was transferred to the ED.

On assessment at the ED, her SpO_2_ had increased to 92% on RA. She was initially given 1 L/minute oxygen via nasal cannula, however, this was successfully weaned after an hour when her saturations had risen to 95% on room air. Her observations were otherwise stable. She had normal regular heart sounds, and the bibasal crepitations persisted. A chest radiograph was consistent with pulmonary oedema (Figure 1(c)). An ECG revealed sinus rhythm with partial left bundle branch block. A further 40 mg of oral furosemide was administered. Her D-dimer was 995 ng/L, and the initial high sensitivity troponin I was 1118 ng/L.

She was admitted overnight. Repeat troponin after 5 hours rose to 2241 ug/L, and a repeat ECG showed inversion of the anterior lead *T* waves. She was reviewed by the on-call cardiology team who recommended starting dual antiplatelet therapy with aspirin and clopidogrel, in addition to ramipril, bisoprolol, and spironolactone given the findings of reduced ejection fraction (∼35%) and anterior/anteroseptal akinesia of the left ventricle on bedside echo; the right ventricle was found to be normal in size and function and was mild to moderate functional mitral regurgitation but,otherwise, normal valves with normal atrial size. She remained stable and pain free overnight and was weaned off supplementary oxygen.

**Figure 2:**
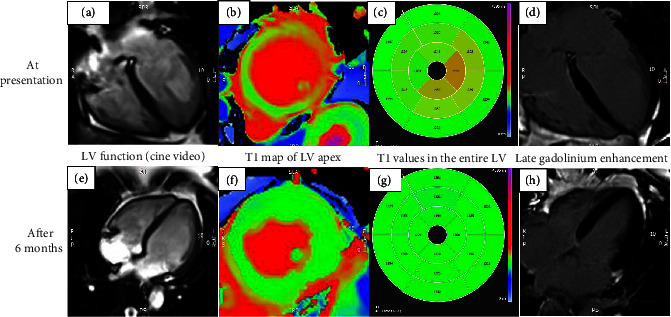
Cardiovascular Magnetic Resonance imaging (CMR) of case 3 performed just prior to the coronary angiogram showed classical features of TCM. There was severe hypokinesia ((a)) and evidence of myocardial oedema on T1 map ((b) and (c)) of mid to apical regions of the left ventricle. The LV ejection fraction was moderately impaired at 43% and the mean global T1 time was elevated at 1311ms. Normal T1 time for a female in 3 Tesla scanner is 1151-1251ms. Late Gadolinium Enhancement (LGE) imaging confirmed the absence of myocardial infarction or fibrosis ((d)). CMR at 6 months shows complete resolution of the regional wall motion abnormalities ((e)) and myocardial oedema ((f) and (g)). The LVEF was much improved to 56% and the mean global T1 time became normalised to 1192 ms with no significant regional differences. Once again, there was no evidence of any myocardial infarction nor fibrosis on LGE imaging ((h)).

On day 2, cardiovascular magnetic resonance (CMR) imaging showed classical features of TCM including severe hypokinesia and myocardial oedema (Figure 2). An invasive coronary angiogram (performed on day 2 of the admission) revealed unobstructed coronary arteries and therefore DAPT and spironolactone were stopped, but bisoprolol and ramipril were continued. A diagnosis of TCM was made, possibly provoked by immersion pulmonary oedema. She was advised to make a slow and gradual return to activity.

## 4. Discussion

This case series reports the simultaneous presentation of SIPE and TCM in three triathletes. There are a limited number of cases of TCM presenting after water immersion (see Table 2) and an even smaller number of reported cases with both SIPE and TCM [14, 15, 20]. The exact pathophysiology of both conditions remains unknown. It is unclear whether the myocardial dysfunction in the presented cases contributed to the causation of SIPE or was the result of SIPE. The clinical presentation, investigation findings, subsequently management with comparisons to both conditions are discussed below.

In the field, SIPE classically presents with acute dyspnoea following water immersion, often accompanied with cough and/or haemoptysis. TCM presents with chest pain more frequently than dyspnoea. All cases in this report had acute dyspnoea with bibasal crackles on lung auscultation. Patients 1 and 3 had associated haemoptysis, and patients 2 and 3 were initially hypoxic. Prompt accurate measurement of oxygen saturations in the prehospital setting is vital and SpO_2_ ≤95% have been shown to be strongly predictive of pulmonary oedema [21]. In combination with crackles on auscultation, this has been shown to have specificity of 98%. Many cases of SIPE improve within hours of leaving the water, which would be quicker than expected if the aetiology was TCM, although TCM can also improve rapidly. An emotional, physical, or combined trigger commonly precedes TCM. It is hypothesized that a catecholamine surge plays a causal role [22]. A mass participation triathlon could provide this trigger. Case 2 in our series admitted feeling anxious prior and this was her first triathlon. However, case 1 was a seasoned triathlete familiar with race stresses.

Whilst the pathophysiology of SIPE remains elusive, it appears that the condition has a multifactorial aetiology with several predisposing factors. In older athletes, episodes of SIPE have been found to commonly unmask underlying pathology, particularly of cardiac cause [10]. This finding does not appear to be true for younger athletes. Risk factors include cold water immersion, overhydration, wetsuit use, hypertension, and female gender [23, 24]. All three cases described, occurred in older athletes swimming in cold, open-water, which likely predisposed them to developing SIPE. Increased preload caused by physical pressure of water immersion, cold-temperature-induced peripheral vaso-constriction, and the compression of a tight-fitting wetsuit increase central venous pressure and may contribute to the development of this syndrome [2]. Age and female gender have also been previously shown to be significant risk factors for SIPE, thought to be caused by age-related relatively increased ventricular elastance/diastolic dysfunction in women and hypertension and myocardial stiffness in both genders [1]. Targeted education in at-risk groups may therefore be beneficial in promoting earlier presentation to event medical services and help to manage the small associated risks of competing in triathlons with advancing age.

Similarly, the exact aetiology of TCM is not fully understood, proposed mechanisms include multivessel coronary artery spasm, impaired cardiac microvascular function, and endogenous catecholamine induced myocardial stunning and microinfarction [25]. The underlying pathophysiology of Takotsubo cardiomyopathy remains poorly understood, but it is recognised that the improvement in left ventricular function usually takes days or weeks with supportive medical care. We agree that this is a result of myocardial injury, but feel that the term infarction/microinfarction is misleading, as the ventricular muscle recovers with no evidence of long-term scarring or infarction. TCM tends to affect women more frequently than men, particularly middle-aged and postmenopausal women [26]. Additional risk factors for TCM include smoking, alcohol abuse, cocaine, anxiety states, and hyperlipidemia [27]. Apart from age and female gender in cases 2 and 3, none of our cases had other underlying risk factors.

CXR remains the mainstay investigation for detecting pulmonary oedema. Point-of-care ultrasound (POCUS) of the lung has been used to verify pulmonary oedema in the field [21],and in conjunction with a field echocardiography to evaluate cardiac function, this may help confirm the isolated diagnosis of SIPE. Nevertheless, those who suffer with an episode of SIPE should have prompt evaluation of their cardiac and pulmonary physiology to rule out coronary disease or structural heart disease causing pulmonary oedema [10, 23].

In contrast to SIPE, TCM produces dramatic electrocardiographic and echocardiographic changes, which resolve in days, weeks, or months. It is usually diagnosed due to the finding of unobstructed coronary arteries with a corresponding wall motion abnormality (typically apical ballooning, but occasionally as a mid-wall or basal variant) and a typical history including a stressful precipitant. The wall motion abnormality can be identified on invasive left ventricular angiography or on echocardiography. Cardiac MRI scanning is emerging as an additional diagnostic tool to identify likely TCM, and typically, this identifies myocardial oedema with no evidence of infarction. TCM is also associated with a troponin elevation as a result of myocardial strain/injury. The magnitude of this elevation can be variable between patients and is likely proportional to the degree of left ventricular impairment. Elevations in troponin I and troponin *T* can be seen (it is also the case that troponin *T* concentrations can rise with extreme exercise due to its presence in skeletal muscle) [28].

The mainstay treatment of SIPE involves safe water evacuation and supplementary oxygen in the prehospital setting. There is often a rapid resolution of signs and symptoms within 48 hours, which can be initiated immediately following removal from water [29]. In case 1, as the patient deemed himself capable of continuing, he did not make himself known to medical staff until after the event and thus was not evacuated from the water; although in this instance no lasting harm was caused. Another episode could potentially be disastrous, and this demonstrates the importance of athlete awareness of SIPE. Effective management also includes advice regarding the return to activities and the risk of recurrence. Whilst the prognosis is generally very good, recurrent episodes are unpredictable. Athletes with a history of SIPE should be presumed to have a predisposition to recurrence, especially if they are found to have an underlying cause. Swimming in controlled environments, starting swimming at a slower pace and prophylactic sildenafil have all been suggested as methods for reducing recurrence rate [8, 23].

Treatment for TCM is largely supportive/symptomatic until left ventricular function is gradually restored. The overall prognosis of TCM remains excellent, with a full recovery observed in 96% of cases [30], though a recent study suggested a risk of recurrence of 7.5% (39/519) [31]. For caution or in more adverse cases, medication including ACE inhibitors (e.g., ramipril) and *ß*-blockers (e.g., bisoprolol) can be used for heart muscle recovery. In all three cases, the athletes were started on long-term ramipril. Despite the largely excellent prognosis, serious complications do occur, including ventricular rupture, thrombosis, arrhythmia, and chronic heart failure, which is why recognising TCM and monitoring these patients are crucial.

The association between SIPE and TCM appears to be two-way, i.e., either condition may precipitate the other. Only a few reports have drawn a direct association between these two specific clinical entities and postulate SIPE as the physical trigger inducing TCM in three cases [12, 24]. It has been suggested that cold water immersion can induce new onset decrease in LVEF and transient apical wall motion abnormalities in women with previous episodes of TCM, as well as increased catecholamine release in control subjects [32]. The emotional and physical stress of the event and the sympathetic stress of SIPE could be compounded by cold water stress, a putative mechanism by which TCM could be triggered in our cases [32]. Given, however, that SIPE is commonly a manifestation of an underlying pathology in the older athlete, it may well be the case that TCM can acutely precipitate SIPE. Two of the case series presented with chest pain which suggests that TCM was the predisposing condition and not a symptom classically associated with SIPE. The strain on the LV together with the supine position held in swimming could increase the likelihood of TCM presenting with pulmonary oedema. However, it is usually seen when LV function is severely impaired.

## 5. Conclusion

Here, we have presented a case series of triathletes diagnosed with SIPE and TCM following an open water swimming event. It is unclear whether the myocardial dysfunction in the presented cases contributed to causation of SIPE or was the result of SIPE. Race organizers and medical teams must be aware and prepared that both SIPE and TCM can present in this group. Those presenting with an episode of SIPE require prompt evaluation of their cardiac and pulmonary physiology.

### 5.1. Practical Implications

- The incidence of SIPE and TCM is likely to increase with the growing popularity of open-water swimming and similar water-based activities.
- Increased awareness and recognition of this clinical entity amongst pre-hospital care workers can assist in prompt diagnosis and accelerate appropriate investigation so that optimum management can ensue.
- More research is required to elucidate the exact physiological relationship between the two conditions.

## Figures and Tables

**Table 1:** A summary of cases presentation, investigation, and management.

	Case 1	Case 2	Case 3
Gender	Male	Female	Female
Age	53	57	60
Race distance	Sprint distance	Sprint distance	Sprint distance
Wetsuit	Yes	Yes	Yes

Initial presentation	Acute dyspnoea	Acute dyspnoea	Severe dyspnoea
	Chest pain (band-like)	Chest pain	Haemoptysis
	Haemoptysis		

Field investigations	SpO_2_: 95% on RA	SpO_2_: 78% on RA	SpO_2_: 88% on RA
	BP: 114/74	BP: 144/80	BP: 112/85
	RR: 26	RR: 34	RR: 20
	HR: 122	HR: 124	HR: 105

Observations at ED	Sats: 97% on 4 L O_2_	Sats: 94% on RA	Sats: 92% on RA and 95% on 1 L O_2_
	BP: 124/86	BP: 111/74	BP: 101/67
	RR: 17	RR: 14	RR: 19
	HR 54	HR: 82	HR: 106

Examination	Bilateral coarse crackles	Bibasal crepitus	Bibasal coarse crackles/creps
	No peripheral oedema		No peripheral oedema

Management at ED	Aspirin 300 mg	Admitted to medical team	1 L O_2_ via nasal cannulae
	Clopidogrel	300 mg oral clopidogrel	30 mg IV furosemide + further 40 mg oral
	GTN 400 mg (sublingual)		DAPT (until normal angiogram confirmed)
	Admitted to medical team		

CXR	Bilateral pulmonary oedema in the hilar region	Pulmonary oedema with upper zone diversion	Bilateral pulmonary oedema with upper zone diversion

Relevant laboratory blood tests:	Troponin: 0.11 ug/L initially and 0.15 at 4 hrs	Troponin: 0.26 ug/L initially and peaked at 1.12 ug/L	Troponin: 1118 ng/L and 2241 ng/L on repeat
	D-Dimer: 1.949 ng/mL	No D-dimer undertaken	No D-dimer undertaken

ECG	*T* wave inversion in V4-6 (nondynamic)	*T* Wave inversion V4-6	*T* Wave inversion in V1-4 (anterior)
			Partial left bundle branch block?

CTPA	No evidence of PE	Not performed	Not performed

TTE	Apical anterior hypokinesia	LV apical ballooning with significant akinesis	Anterior and anterioseptal akinesis
	“Good biventricular function”	Good RV function	Mild LV dilation
			LVED 35%
			Mild mitral regurg
			Good RV function

CMR	N/A	N/A	Classical TCM features (see figure).
			LVEF 43%.

Angiogram	Mild nonobstructive atheroma in mid-LAD	Normal coronary arteries	Normal coronary arteries

Management admission-onwards:	GTN administered	Started on aspirin, ramipril 1.25 mg qd bisoprolol 1.25 mg qd	Started on:
	Started on:		Ramipril 1.25 mg qd
	Aspirin 75 mg,		Bisoprolol 2.5 mg qd
	Atorvastatin 40 mg,		
	Ramipril 1.25 mg qd		

Lifestyle advice	No exercise for 6 weeks	Refrain from strenuous activities for the next 3 months	Slow and gradual return to activity

ED, emergency department; CXR, chest radiograph; ECG, electrocardiogram; CTPA, CT pulmonary angiography; TTE, transthoracic echocardiogram.

**Table 2:** Report case of swimming related case of TCM.

Author	Case	Age (gender)	Context	Presenting symptoms	Echo summary: key findings	Other markers?
De Gennaro et al. [13]	1	77 (F)	Sea swimming rescued by a life-guard following drifting out to sea	Acute dyspnoea and Chest pain	LVEF: 25% Apical dyskinesis and basal hyperkinesis resembling apical ballooning	Angio: normal coronary arteries

Reed [14]	3	62 (F)	Scuba diver	Acute dyspnoea & cough & frothy pink sputum	LVEF: 45% (later improved to 55%) Apical ballooning with basal hyperkinesis	Angio: normal coronaries Troponin I: peaked at 0.84 ug/L ECG: deep pathological *Q* waves (V1-3), *T* wave inversion (I, avL, V6)
		60 (M)	Scuba diver	Acute dyspnea and foamy brown sputum	LVEF: 25% Global hypokinesis with apical ballooning	Angio: normal coronaries Troponin I: 0.53 ug/L (peak) CXR: pulmonary oedema suggestive of congestive heart failure
		47 (F)	Scuba diver	Tachypnea	LVEF: 45% Diminished left ventricular compliance Anterior and inferior severe hypokinesis	Angio: normal coronaries O_2_ sat: 88% on room air Troponin I: 3.49 ug/L CXR: bilateral moderate infiltrates

Chenaitia et al. [15]	1	51 (F)	Scuba diver	Acute dyspnea Chest tightness	LVEF: 35% Left ventricular hypokinesia, apical ballooning and basal hyperkinesia	Angio: normal coronaries CXR: bilateral infiltrates of lower lungs Bilateral pulmonary creps up to the midfield O_2_ sat: 100% on 15 L ECG: left axis deviation

Citro et al. [16]	1	53 (F)	Open water swimming	Acute dyspnea Tachypnea	Apical ballooning Left ventricular hyperkinesis	Angio: normal coronaries Troponin I: increased

Baber et al. [17]	1	47 (F)	Scuba diver	Acute dyspnea Tachypnea	Left ventricular basal hypokinesis and apical hyperkinesis	Angio: mild, nonobstructive coronary artery disease CXR: pulmonary oedema Troponin 1: 1.10 ug/L (peak) ECG: sinus tachycardia with mild ST depressions in the inferior and anterolateral leads

Beinart et al. [18]	1	54 (F)	Cold water swimming	Acute dyspnea	Global severe left and right ventricular dysfunction	Angio: normal coronaries Troponin I: 11 ug/L O_2_ sat: 64% on air in field ECG: minimal ST depression in leads I and aVL

Dessardo et al. [19]	1	12 (F)	Sea water swimming	Acute dyspnea Chest pain Cyanosis	Angio: normal coronaries LVEF: 30–32% Left ventricular hypokinesia	Troponin I: 1.53 ug/L ECG: acute ischaemic lesions in the anterioseptal leads

## Data Availability

The data used to support the findings of this study are included within the article.
