# Erratum to “Case Series of Triathletes with Takotsubo Cardiomyopathy Presenting with Swimming-Induced Pulmonary Edema”

**DOI:** 10.1155/2023/9858459

**Published:** 2023-10-11

**Authors:** Caitlin Rigler, Gautam Menon, Samuel Lipworth, Jeremy P. Langrish, Courtney Kipps, Mayooran Shanmuganathan, Ralph Smith

**Affiliations:** ^1^Department of Sport and Exercise Medicine, Nuffield Orthopaedic Centre, Oxford University Hospitals NHS Trust, Oxford, UK; ^2^Emergency Department, John Radcliffe Hospital, Oxford University Hospitals NHS Trust, Oxford, UK; ^3^Department of Cardiology, John Radcliffe Hospital, Oxford University Hospitals NHS Trust, Oxford, UK; ^4^Institute of Sport, Exercise and Health, Division of Surgery and Interventional Sciences, University College London, London, UK

In the article titled “Case Series of Triathletes with Takotsubo Cardiomyopathy Presenting with Swimming-Induced Pulmonary Edema” [1], there was an error in Figure 1.

The corrected figure is shown as follows and is listed as Figure 1.

The error was introduced during the production process of the article, and Hindawi apologies for causing this error in the article.

## Figures and Tables

**Figure 1:**
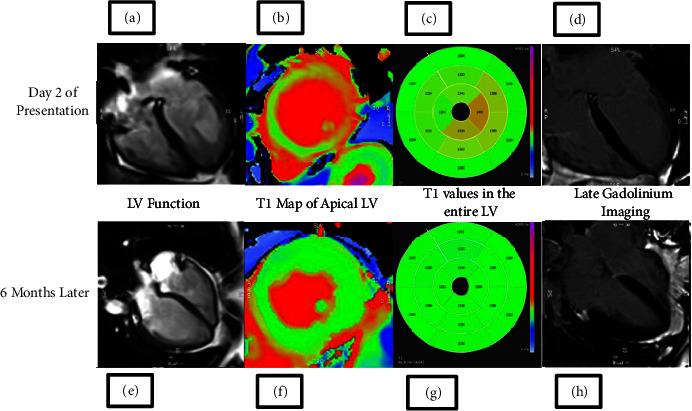
Cardiovascular Magnetic Resonance imaging (CMR) of case 3 performed just prior to the coronary angiogram showed classical features of TCM. There was severe hypokinesia (a) and evidence of myocardial oedema on T1 map (b and c) of mid to apical regions of the left ventricle. The LV ejection fraction was moderately impaired at 43% and the mean global T1 time was elevated at 1311 ms. Normal T1 time for a female in our 3 Tesla scanner is 1151–1251 ms. Late Gadolinium Enhancement (LGE) imaging confirmed the absence of myocardial infarction or fibrosis (d). CMR at 6 months shows complete resolution of the regional wall motion abnormalities (e) and myocardial oedema (f and g). The LVEF was much improved to 56% and the mean global T1 time became normalised to 1192 ms with no significant regional differences. Once again, there was no evidence of any myocardial infarction nor fibrosis on LGE imaging (h).
